# Bursal-Derived BP7 Induces the miRNA Molecular Basis of Chicken Macrophages and Promotes the Differentiation of B Cells

**DOI:** 10.3390/vaccines10111960

**Published:** 2022-11-18

**Authors:** Jiaxi Cai, Ze Zhang, Chenfei Li, Shanshan Hao, Anran Lu, Xiangyu Huang, Xiuli Feng

**Affiliations:** 1 Key Laboratory of Animal Microbiology of China’s Ministry of Agriculture, College of Veterinary Medicine, Nanjing Agricultural University, Nanjing 210095, China; 2 MOE Joint International Research Laboratory of Animal Health and Food Safety, College of Veterinary Medicine, Nanjing Agricultural University, Nanjing 210095, China

**Keywords:** BP7, chicken macrophages, immune induction, B cell differentiation

## Abstract

The bursa of Fabricius (BF) is a vital central humoral immune organ unique to birds. The bioactive peptide BP7 derived from bursa is reported to promote the vaccine immune response and antibody production. However, the regulatory effect on antigen presentation and B cell differentiation has been infrequently reported. In this paper, chicken macrophage HD11 cells were used for the cell model, and the cellular molecular expressions were determined by the fluorescent quantitative PCR (qPCR) after BP7 treatment. Then, the miRNA expression profile was analyzed by high-throughput sequencing. In addition, BALB/C mice were used as the animal model to detect B cell subtype with flow cytometry (FCM). The results showed that the expressions of four immune active molecules, IL-1β, IL-6, iNOS, and IFN-α, in HD11 cells were significantly increased with 100 ng/mL BP7 treatment. Compared with the control group, there were 58 up-regulated and 61 down-regulated miRNAs in HD11 cells with BP7 treatment. The gene ontology (GO) function analysis found that BP7 mainly affected the various biological processes, molecular function, and MHC protein complex. Pathway analysis showed that 100 ng/mL BP7 stimulated various physiological metabolic pathways and signal transduction pathways, including the intestinal immune network producing IgA in HD11 cells. Furthermore, it was found that BP7 in vitro stimulated B cell populations, as well as plasma cells in spleen cells from the immunized mice. Additionally, B cell activation subpopulations were increased in mice immunized with the AIV vaccine and BP7. These results proved that BP7 stimulated various differentially expressed genes in chicken macrophage HD11 cells, and induced B cell differentiation in the immunized mice, which suggested that BP7 might participate in the antigen presentation process, thereby promoting the differentiation of B cells. These results provide an important basis for the mechanism of bursal-derived peptide on B cell development, and offer the experimental basis for the development of adjuvants.

## 1. Introduction

The bursa of Fabricius (BF) is a well-known humoral central immune organ unique to birds, which is crucial to the differentiation of B lymphocytes and immune development [1,2,3]. At first, it is wrongly thought to be related to the reproductive function [4], but later, it is proven that BF is closely related to the antibody formation and immunity [5,6,7,8]. The active peptides are the important active components of BF, which can regulate the humoral immunity and B cell differentiation [9,10,11]. They also have various functions, inducing antioxidation [12,13] and anti-tumor effects [14,15,16]. The functional mechanism of bursal-derived peptides on regulation of immunity and antigen presentation is worthy of further study.

Avian influenza is an acute and highly contagious disease caused by avian influenza A virus (AIV). The H9N2 subtype AIV is equally likely to be infected in all domestic and wild birds [17,18]. It is also reported that H9N2 AIV may be a potential threat to human health [19]. Vaccination has become a key measure to prevent diseases and protect the health of livestock and poultry against AIV infection [20,21]. In addition to the relevant antigen components, the appropriate adjuvant for the vaccine is a basic requirement for promoting immune response. MicroRNA (miRNA) is a non-coding short RNA molecule with a length of 19–25 nucleotides [22], which plays an indispensable role in the immune system [23]. Avian macrophages are the key regulatory cells of the immune system, which initiate and direct the innate and specific immune responses in chickens [24]. It is very important to study the molecular mechanism of miRNA expression profiles in chicken macrophages with the immunoenhancer for the prevention and control of avian influenza.

The bursal-derived active peptide BP7 has recently been reported to promote vaccine immune response in chicken immunization [25], but the molecular basis of on miRNA expression profile of antigen presentation and immune function of BP7 inducing chicken macrophages has not been reported. In order to investigate the mechanism of bursal-derived peptides on antigen presentation and B cell development, in this study, the chicken macrophage was used as the cell model to study the effect of BP7 on the expression of immune active molecules. Then, the miRNA expression profile of chicken macrophages stimulated by BP7 through high-throughput sequencing technology was analyzed. Finally, the H9N2 AIV vaccine was used to immunize mice, and the effect of in vitro stimulation and in vivo immunization of BP7 on B cell differentiation was explored. These studies will provide the basis for the clinical application potential of BP7 in promoting chicken vaccine immunity, and offer the support for the prevention and control of avian influenza.

## 2. Materials and Methods

### 2.1. Peptide Synthesis, Cells and Animals

BP7, with seven amino acids of GGCDGAA, was synthesized by GenScript Biotech Corp (Nanjing, China) with a purity of 95%. Chicken macrophage HD11 cells were cultured in DMEM medium (Gibcol) with 10% fetal bovine serum (Wisent, Nanjing, China). 4–6-week-old BALB/c mice were purchased from the Experimental Animal Center of Yangzhou University (Yangzhou, China).

### 2.2. MTT Assay

HD11 cells were treated with BP7 at dosages from 0.01 to 100 µg/mL for 48 h, and then incubated with 20 µL 5 mg/mL MTT for 6 h. Additionally, DH11 cells were treated with 10 ng/mL BSA to be used as control. After discarding the culture medium, 100 μL DMSO was added into the cell in order to lyse cells, and then the value under the condition of 570 nm absorbance was read to analyze the viability of HD11 cells.

### 2.3. Determination of Cytokine Expression in HD11 Cells Treated with BP7

HD11 cells were treated with 10, 100, and 1000 ng/mL BP7 and 10 ng/mL BSA, in which BSA was used as control. At 6, 12, 24, and 48 h after treatment, the total RNAs from the cell samples were collected in order to detect the mRNA expressions of IL-1β, IL-6, INOS, IFN-α, and IL-10 cytokines with the qPCR [26]. β-actin was used as the internal reference gene. The primers for these cytokines were listed in Table 1.

### 2.4. Preparation of Sequencing Samples and High Throughput miRNA Sequencing

**Figure 1:**
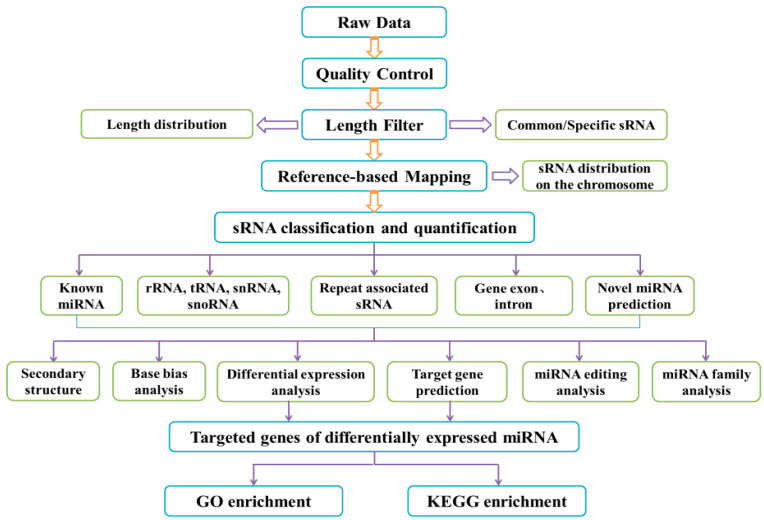
Schematic diagram of the biological information analysis in HD11 cells with BP7 treatment.

HD11 cells were treated with 100 ng/mL BP7 and 10 ng/mL BSA for 48 h, and the total RNAs of the treated HD11 cells were collected with Trizol (Takara, Japan). The high throughput sequencing and library construction of miRNA were completed by Novogene Company (Beijing, China). Simply, after quality detection of the RNA sample, the cDNA library was constructed following the small RNA sample pre kit and sequencing. In addition, based on the reference genome of the chicken species, the biological information was analyzed following the schematic diagram (Figure 1). Based on GO and the Kyoto encyclopedia of genes and genomes (KEGG) pathway database, the differentially expressed genes (DEGs) were compared and analyzed with a *p*-value of less than 0.05.

### 2.5. Mice Immunization Model

4–6-week-old BALB/c mice, as the immunization model animals, were listed as follows. In the first model, mice were intraperitoneally injected with AIV antigen plus oil adjuvant three times, with an interval of two weeks. On the seventh day after the third immunization, the spleen cells were collected from the immunized mice and stimulated with 10, 100, and 1000 ng/mL BP7 for 48 h. The LPS-stimulated group was used as the positive control, and BSA treatment was used as the control. The spleen cells were incubated with the fluorescent labeled antibody to CD19, CD43, CD27, and CD38, and B cell subtypes were detected with FCM.

In the second model, mice were intraperitoneally injected with AIV antigen plus oil adjuvant and 0.01, 0.05, and 0.25 mg/mL BP7 two times, at an interval of two weeks. At one week after the second immunization, the serum samples were collected from the immunized mice to detect the antibody levels. Spleen cells of the immunized mice were cultured in DMEM medium for 48 h, and treated with MTT to detect the spleen cell viabilities. In addition, the spleen cells were collected, and then incubated with the fluorescent labeled antibody to CD19, CD69, and CD38 to detect the B cell subtypes with FCM.

### 2.6. Data Analysis

All of the data were counted, and Graphpad Prism 5.0 (GraphPad software, San Diego, CA, USA) was used to analyze the data. The data were shown as mean value ± standard deviation (S.D.). Significant differences between groups were determined using Student’s *t*-test.

## 3. Results

### 3.1. The Viabilities of HD11 Cells with BP7 Treatment

**Figure 2:**
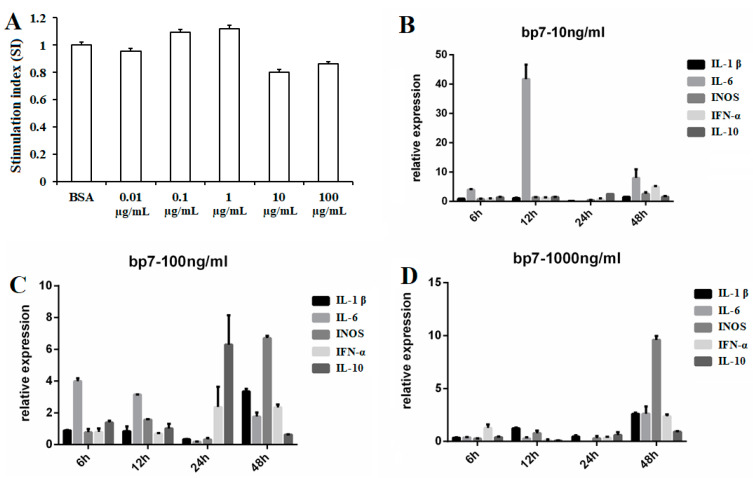
The viabilities and cytokine expressions in HD11 cells with BP7 treatment. HD11 cells were treated with BP7, and the proliferation and the mRNA expressions of five cytokines were detected. (**A**)The viabilities of HD11 cells. (**B**) The cytokines expression in HD11 cells treated with 10 ng/mL BP7. (**C**) The cytokine expression in HD11 cells treated with 100 ng/mL BP7. (**D**) The cytokine expression in HD11 cells treated with 1000 ng/mL BP7.

HD11 cells were treated with BP7 for 24 h, and the viabilities of HD11 cells were detected with MTT assay. The results showed that the viabilities of HD11 cells with 0.01, 0.1, and 1 µg/mL BP7 were similar to that of the control, with no significant difference (Figure 2A). However, compared to that of the control, the viabilities of HD11 cells treated with 10 and 100 µg/mL BP7 were decreased.

### 3.2. The Expressions of the Cytokine in HD11 Cells with BP7 Stimulation

In order to explore the regulatory function of BP7 on chick macrophages, HD11 cells were stimulated with 0.01, 0.1, and 1 µg/mL BP7, and the mRNA expressions of five representative cytokine were detected with qPCR at a different time. The results showed that while the expression of IL-6 in HD11 cells at 12 h increased, 0.01 µg/mL BP7 did not stimulate HD11 cells to produce the five cytokines (Figure 2B). It was observed that 0.1 µg/mL BP7 significantly enhanced the expressions of the five cytokines in HD11 cells at 6, 12, 24, and 48 h after BP7 treatment, and that the expressions of the five cytokines at 48 h were most significant (Figure 2C). Additionally, the expressions of the five cytokines in HD11 cells at 48 h after 1 µg/mL BP7 treatment were significantly increased (Figure 2D), whereas the cytokine expressions in HD11 cells treated with 1 µg/mL BP7 at other experimental points in time were not significantly different. Therefore, the samples of HD11 cells treated with 0.1 µg/mL BP7 for 48 h were used in the subsequent experiment.

### 3.3. Library Construction Analysis

The total RNAs of HD11 cells treated with 0.1 µg/mL BP7 for 48 h were collected in order to employ the high throughput miRNA sequencing. The results showed that the RIN values of small RNA from BP7 treatment and BSA control were 10 (Table 2), which suggested that these samples were qualified and the library could be built normally.

**Figure 3:**
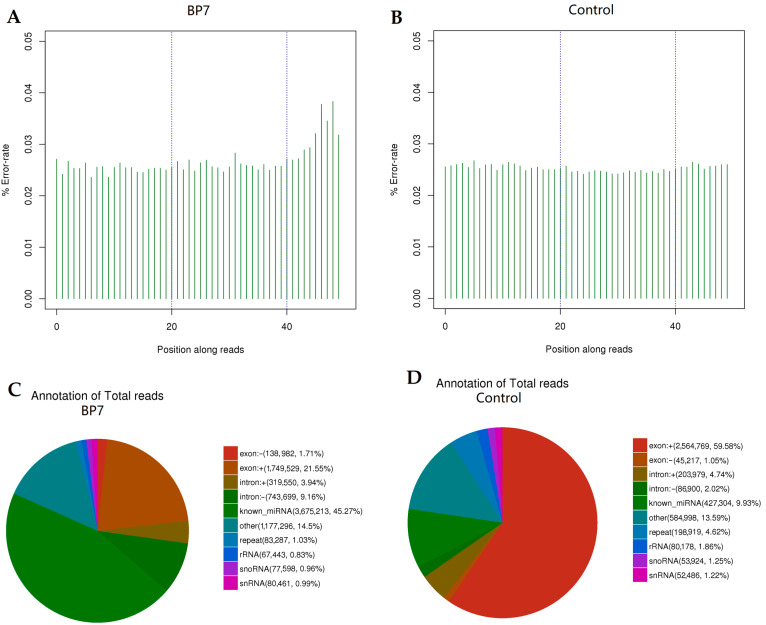
**The error rate distribution and RNA category in HD11 cells with BP7 treatment.** HD11 cells were treated with 100 ng/mL BP7 for 48 h, and HD11 cells treated with BSA were used as control. (**A**) The error rete distribution of HD11 cells with BP7 treatment. (**B**) The error rete distribution of HD11 cells with BSA treatment (control). (**C**) The RNA category in HD11 cells with BP7 treatment. (**D**) The RNA category in HD11 cells with BSA treatment (control).

In addition, the original data error rate distribution was checked, as showed in Figure 3A,B. The sequencing error rates of the BP7 stimulated sample and its control were lower than 0.5%, indicating that the sequencing results were good.

The RNA types in the all small RNAs analyzed from the HD11 cells treated with BP7 and control were compared, and the annotations are summarized in Figure 3C,D. Generally, the proportion of total rRNA in animal samples with good quality should be less than 40%. The results showed that the rRNA ratios of BP7 treatment and control were lower than 40%, indicating that the total rRNA samples of BP7 treatment and control were of good quality (Figure 3C,D). Compared with the control group, the proportion of known miRNAs in the BP7 stimulated group was significantly increased, while the proportion of exons was decreased, and other types of sRNAs showed no significant difference.

### 3.4. miRNA Expression Profile Analysis

**Figure 4:**
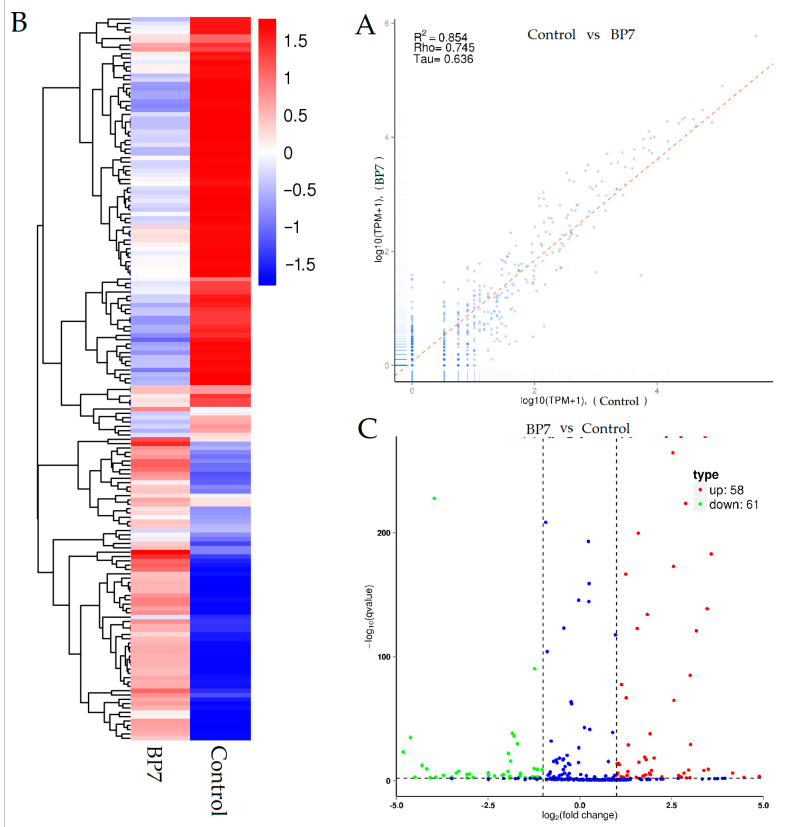
**The miRNA expression profile analysis and validation in HD11 cells with BP7 treatment.** HD11 cells were treated with BP7 and BSA for 48 h, and the miRNA expression profile were analyzed. (**A**) The expression quantity correlation scatter plot of HD11 cells with BP7 and BSA treatment. (**B**) The miRNA expression profiles in HD11 cells with BP7 and BSA treatment. (**C**) The differentially expressed miRNAs in HD11 cells with BP7. The blue points indicated the non-differentially expressed miRNAs.

The correlation of microRNA expression levels between BP7 and control is an important indicator to test the reliability of experiments and the rationality of sample selection. The expression quantity correlation scatter plot is displayed in Figure 4A. The correlation coefficient was 0.854, suggesting that the expression pattern between BP7 and control was of high similarity. The fold change and q value were used to evaluate the expressed different miRNA, whose heat map analysis is shown in Figure 4B. It was observed that compared with the control, BP7 stimulated 119 differentially expressed miRNAs in HD11 cells, including 8 up-regulated miRNAs and 61 down-regulated miRNAs. The overall distribution of differential miRNAs is listed in the volcano map (Figure 4C).

### 3.5. The Biological Function Analysis in HD11 Cells with BP7 Treatment

**Figure 5:**
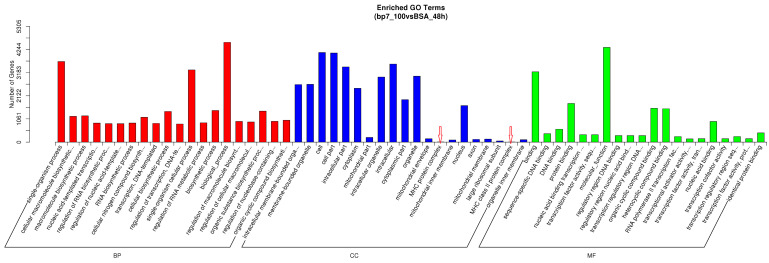
**The biological function analysis in HD11 cells with BP7 treatment.** The biological function analysis was divided into three classifications, namely, biological processes (BP), cellular component (CC), and molecular function (MF). The top 20 with the largest number of differentially expressed genes in the three categories were listed.

In order to explore the biological function of BP7 on chicken macrophages, in this paper, we first analyzed the enriched GO terms with the number within the top 20 in biological process (BP), cellular component (CC), and molecular function (MF, Figure 5), respectively. The results showed that the number of DEGs of the biological processes and single-organism process were the highest among BP terms, and the binding and molecular function were the highest among MF terms. In addition, cell and cell part terms of CC were found to have a large number of DEGs, and the MHC protein complex and the MHC class II protein complex were the enriched terms.

In order to further investigate the molecular basis of BP7 on the immune response, the immune-related GO terms in HD11 cells with BP7 treatment were analyzed and summarized in Table 3, which included immune, MHC, and antigen presentation, as well as B cell- and cytokine-related GO terms. These results suggested that BP7 might induce various immune-related biological processes and functions.

### 3.6. The Enriched Pathway in HD11 Cells with BP7 Treatment

In order to investigate the molecular mechanism of BP7 on chicken macrophages, in this paper, the enriched pathway in HD11 cells with 0.1 µg/mL BP7 treatment was analyzed. The results showed that BP7 induced various DEGs involved in six enriched pathways (Table 4), including those of the intestinal immune network for IgA production, fructose and mannose metabolism, endocytosis, ribosome, glycosylphosphatidylinositol (GPI)-anchor biosynthesis, and cell adhesion molecules (CAMs). These enrichment pathways involved the immune response, physiological metabolism, biosynthesis, and adhesion factors, which suggested that BP7 might induce the multiple biological functions to regulate the immune response.

### 3.7. BP7 Induced B Cell Differentiation In Vitro

**Figure 6:**
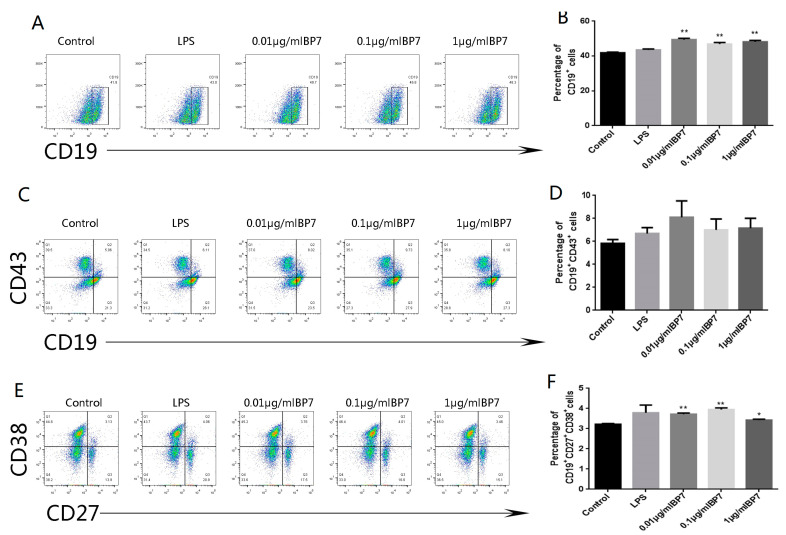
**B cell subtype with BP7 treatment in vitro.** The spleen cells were collected from the mice immunized with the H9N2 AIV vaccine, and were stimulated with BP7 at three dosages for 48 h. BSA treatment was used as control. (**A**) Flow cytometry plot of CD19+ B cells with BP7 treatment. (**B**) Histogram of CD19+ B cells percentage with BP7 treatment. (**C**) Flow cytometry plot of CD19+CD43+ B cells with BP7 treatment. (**D**) Histogram of CD19+CD43+ B cell percentage with BP7 treatment. (**E**) Flow cytometry plot of CD27+CD38+ B cells with BP7 treatment. (**F**) Histogram of CD27+CD38+ B cells with BP7 treatment. * *p* < 0.05, ** *p* < 0.01, compared to that of control.

In order to explore the effect of BP7 on B cell differentiation, B cells with a purity of 96.7% were collected from the immunized mice following the magnetic bead separation, and were then stimulated with BP7 for 48 h to detect the subtypes of B cells. The results showed that compared with the control group, BP7 at 0.01, 0.1, and 1 µg/mL significantly promoted the populations of CD19+ total B cells (Figure 6A,B). It was observed that there was little difference among three BP7 concentration stimulation groups. Additionally, it was observed that three concentrations of BP7 significantly enhanced the activation of B cells in the spleen cells from the immunized mice (Figure 6C,D). The population of CD43+ CD19+ B cells with 0.01 µg/mL BP7 treatment was the highest among all the groups, even higher than that in the LPS control. Furthermore, it was found that the populations of CD27+ CD38+ plasma cells were significantly increased with BP7 at three concentrations compared with that of the control (Figure 6E,F), in which the plasma cell level in the 0.1 µg/mL BP7 groups was the highest. These results indicated that BP7 could stimulate the proliferation and activation of total B cells and plasma cells production. However, the relationship between the dosage and the induction effect needed to be further explored.

### 3.8. BP7 Induced B Cell Differentiation In Vivo

The immunized animals might be a better model to reflect the induction of BP7 on the differentiation of B cells.

**Figure 7:**
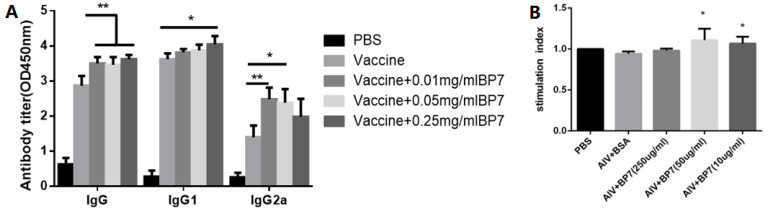
**Antibody production and viabilities in mice immunized with AIV vaccine and BP7.** Mice were twice immunized with the AIV vaccine and BP7 at two week intervals. (**A**) Antibody production of the mice in all experimental groups. (**B**) The viabilities of spleen cells from the mice in all experimental groups. * *p* < 0.05, ** *p* < 0.01, compared to that of the vaccine groups.

In order to study the effects of BP7 on the humoral and B cell immunity, mice were twice immunized with avian influenza vaccine, and with 0.01, 0.05, and 0.25 mg/mL BP7, respectively, and the changes in serums IgG, IgG1, and IgG2a were detected by ELISA. The results showed that compared with the vaccine control, the immunization groups with three doses of BP7 significantly promoted the increase in IgG levels in mice (Figure 7A, IgG). It was observed that the 0.25 mg/mLBP7 group significantly stimulated the increase in IgG1 levels in mice (Figure 7A, IgG1), and that 0.01 mg/mL BP7 and 0.05 mg/mL BP7 also significantly stimulated the increase in IgG2a levels in mice (Figure 7A, IgG2a). In addition, it was found that compared with the AIV vaccine control, the group immunized with 0.05 and 0.01 mg/mL BP7 combined with the AIV vaccine significantly stimulated the activity of splenic lymphocytes (Figure 7B). These results indicated that BP7 might stimulate the antibody immune in the immunized mice.

**Figure 8:**
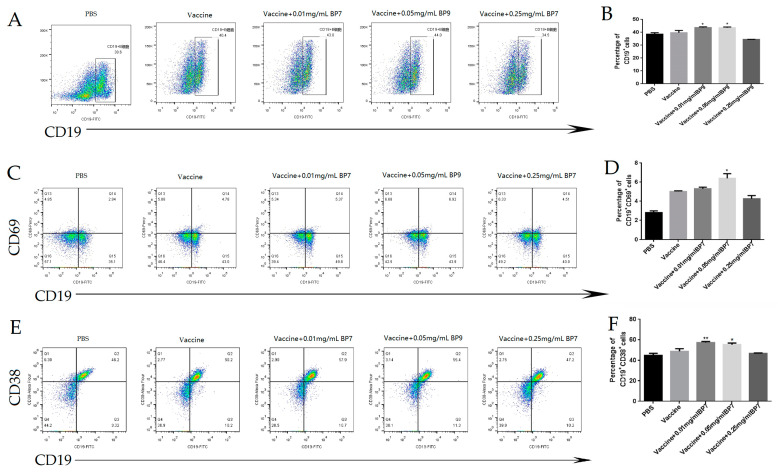
**B cell subtype in spleen cells from the mice immunized with BP7.** Mice were twice immunized with the H9N2 AIV vaccine and 0.01, 0.05, and 0.25 mg/mL BP7. The spleen cells were collected from all groups to detect the subpopulation of B cells. (**A**) Flow cytometry plot of CD19+ B cells of all groups. (**B**) Histogram of CD19+ B cells percentage of all groups. (**C**) Flow cytometry plot of CD19+CD69+ B cells of all groups. (**D**) Histogram of CD19+CD69+ B cells percentage of all groups. (**E**) Flow cytometry plot of CD19+CD38+ B cells of all groups. (**F**) Histogram of CD19+CD38+ B cells of all groups. * *p* < 0.05, ** *p* < 0.01, compared to that of vaccine group.

In this paper, B cell differentiation and activation in the mice immunized with the AIV vaccine and BP7 were detected with FCM. The results showed that compared with the vaccine group, the percentages of CD19+ total B cells in the 0.01 mg/mL and 0.05 mg/mL BP7 combined vaccine experimental groups were significantly increased (Figure 8A,B). Compared with the vaccine group, the number of CD19+CD69+ activated B cells in the 0.05 mg/mL combined vaccine immunized group was significantly increased, and was greater than that of the vaccine group (Figure 8C,D). Additionally, compared with the vaccine group, the percentages of CD19+CD38+ cells in the 0.01 mg/mL and 0.05 mg/mL BP7 combined immunization groups were increased, with the most significant increase of 0.01 mg/mL. However, the percentage of CD19+CD38+ cells in the 0.25 mg/mL BP7 combined immunization group was lower than that of the vaccine group (Figure 8E,F). These results suggested that BP7 might have the dual regulatory effects on B cell differentiation in vivo. A low concentration of BP7 could promote the proliferation and differentiation of B cells, while a high concentration of BP7 might inhibit the proliferation and differentiation of B cells. Thus, the dose of BP7 and activation of B cells still might need to be further explored.

## 4. Discussion

BF is the recognized humoral central immune organ, which provides an ideal research model for studying the functional mechanism of human and mammalian central humoral organs and vaccine development [2]. Bursal-derived active peptides significantly improve the ability of chickens to produce specific antibodies, and have a significant role in promoting the immune system of poultry [1,27]. It is necessary to further study the mechanism of bursal peptides on antigen presentation and B cell development, and it is beneficial to human health when applied to the clinical practice.

Macrophages, which have the function of antigen-presenting cells, are important objects of cellular and molecular immunology [28]. In this study, chicken macrophage HD11 cells were used as a cell model to investigate the function and mechanism of BP7 on chicken macrophages. It was proven that 0.1 µg/mL BP7 promoted the expressions of various cytokines productions and induced the differentially expressions of 119 DEGs following high-throughput technology. In addition, the biological functions of the DEGs in HD11 cells with BP7 stimulation were analyzed based on the ontological function of genes. It was found that BP7 could regulate various biological processes, cellular components, and molecular function terms, in which BP7 could stimulate various immune related terms, including MHC complex, B cell, cytokines, and chemokines. It was reported that during the immune response activated with antigen, MHC molecules play a vital role in the repertoire selection and maintenance, as well as the T/B cell activation and function [29]. The naïve T cells produce multiple cytokines upon activation, while B cells require the specific differentiation and activation conditions to produce cytokines [30]. Chemokine have important functions in the immature B cell development and B cell fate [31]. These results suggested that BP7 might induce various B cell development-related immune functions.

Furthermore, pathway analysis results showed that these differentially expressed genes involved six enriched physiological and metabolic pathways and signal transduction pathways, including the intestinal immune network that produces IgA [32], fructose, and mannose metabolism and endocytosis. The bursa of Fabricius is located at the end of the intestinal system. The gut microbial environment leads to a continuous diversification of B-cell repertoire and the production of antibodies, especially IgA, which provides an elegant educational process for the adaptive immune network. Glycometabolism is reported to regulate the formation, maintenance, and function of memory CD8+ T cells [33]. B cells promote CD8 T cell primary and memory responses to subunit vaccines [34], in which endocytosis plays a role in antigen presentation to T cells [35]. In addition, after antigen capture, B cell antigens internalize and proceed to late endosomes via clathrin-dependent endocytosis and intracellular routing [36]. These results indicated that BP7, as an active molecule derived from avian bursa, might participate in the process of immune function and antigen presentation for immune cells, which would be helpful for the immune response and B cell differentiation.

The B cell is the vital index the immune activation and antigen presentation [37]. In this paper, the inducing functions of BP7 on B cell differentiation in both in vitro and in vivo immunization models were detected. BP7 stimulated the increased subpopulation of CD43+CD19+ and CD27+CD38+ B cells in vitro from the mice immunized with the AIV vaccine, and then enhanced the percentages of CD19+CD69+ and CD19+CD38+ B cells in vivo from the mice immunized with BP7 and the AIV vaccine. Additionally, BP7, with its low molecular weight and simple structure composition, is a small polypeptide. The antigenicity of BP7 is very slight. In the immunization experiment, BP7 was not coupled with carrier, and not polymerized into polymers. No antibody special to BP7 was observed in the immunized mice. These results suggest that BP7, as the immune-enhancing polypeptide, might induce the activation of total B cells and plasma cells production. However, the relationship between the dosage and the inducing function needed to be further explored.

## 5. Conclusions

In conclusion, BP7, the recently reported bioactive peptide derived from bursa, enhances the antibody response during vaccine immunization. In this study, BP7 stimulated the mRNA expression of various cytokines, and induced 58 up-regulated and 61 down-regulated miRNAs in chicken macrophage HD11 cells. These differentially expressed genes were involved in various biological processes, molecular functions and MHC protein complexes, and participated in six physiological metabolic and signal transduction pathways. These results proved the molecular basis of BP7 on immune induction in chicken macrophages. Furthermore, in the BALB/c mice immunization model, BP7 stimulated B cell populations in vitro and enhanced B cell activation in vivo. These results provide an important basis for a mechanism of bursal-derived peptide for B cell development, and offer the experimental basis for the development of adjuvants.

## Figures and Tables

**Table 1:** Primers of Reference Genes and Cytokines in Real-Time PCR.

Primer Names	Primer Sequences
IL-1β-F	ACCCGCTTCATCTTCTACCG
IL-1β-R	TCAGCGCCCACTTAGCTTG
IL-6-F	AGGACGAGATGTGCAAGAAGTTC
IL-6-R	TTGGGCAGGTTGAGGTTGTT
IL-10-F	CGCTGTCACCGCTTCTTCA
IL-10-R	CGTCTCCTTGATCTGCTTGATG
INOS-F	AGGCCAAACATCCTGGAGGTC
INOS-R	TCATAGAGACGCTGCTGCCAG
IFN-α-F	GGACATGGCTCCCACACTAC
IFN-α-R	GGCTGCTGAGGATTTTGAAGA
β-actin-F	AGACATCAGGGTGTGATGGTTGGT
β-actin-R	TGGTGACAATACCGTGTTCAATGG

**Table 2:** Sample quality test results.

Sample	Concentration (ng/µL)	Total RNA (μg)	RIN	Detection
BP7	334	10.688	10.00	A
Control	63	2.016	10.00	A

**Table 3:** Profile of immune-related gene ontology terms in HD11 cells after BP7 treatment.

GO Accession	Description	Term Type	Over_Represented_*p*-Value	DEG Item
Immune-related GO terms				
GO:0002683	negative regulation of immune system process	BP	0.0032905	99
GO:0002376	immune system process	BP	0.0072332	488
GO:0045824	negative regulation of innate immune response	BP	0.030752	10
MHC and antigen presentation-related GO terms				
GO:0042611	MHC protein complex	CC	4.71 × 10^−5^	9
GO:0042613	MHC class II protein complex	CC	0.00014287	8
GO:0042287	MHC protein binding	MF	0.040487	7
GO:0002483	antigen processing and presentation of endogenous peptide antigen	BP	0.046177	4
B cell-related GO terms				
GO:0050869	negative regulation of B cell activation	BP	0.014968	12
GO:0045578	negative regulation of B cell differentiation	BP	0.038157	3
GO:0001922	B-1 B cell homeostasis	BP	0.039526	3
Cytokine-related GO terms				
GO:0001816	cytokine production	BP	0.0028395	148
GO:0071346	cellular response to interferon-gamma	BP	0.016662	31
GO:0034341	response to interferon-gamma	BP	0.029455	32
GO:0070098	chemokine-mediated signaling pathway	BP	0.0014371	29
GO:0048020	CCR chemokine receptor binding	MF	0.0049025	15

Note: BP: biological process, CC: cellular component, MF: molecular function.

**Table 4:** The enriched pathway in HD11 cells with BP7 treatment.

ID	Term	Rich Factor	Number	q Value
gga04672	Intestinal immune network for IgA production	0.6875	22	0.9476629
gga00051	Fructose and mannose metabolism	0.625	20	0.9476629
gga04144	Endocytosis	0.4635417	89	0.9476629
gga03010	Ribosome	0.4833333	58	0.9476629
gga00563	Glycosylphosphatidylinositol (GPI)-anchor biosynthesis	0.625	15	0.9476629
gga04514	Cell adhesion molecules (CAMs)	0.4732143	53	0.9476629

## Data Availability

Not applicable.
